# Characterizing Unique Clinical and Virological Profiles in Concurrent Chronic Hepatitis B and Metabolic Dysfunction-Associated Liver Disease: Insights from a Population-Based Cohort Study

**DOI:** 10.3390/jcm13185608

**Published:** 2024-09-21

**Authors:** Fadi Abu Baker, Abdel-Rauf Zeina, Randa Taher, Saif Abu Mouch, Ariel Israel

**Affiliations:** 1 Department of Gastroenterology and Hepatology, Hillel Yaffe Medical Center, Technion Faculty of Medicine, Hadera 38100, Israel; fa_fd@hotmail.com (F.A.B.);; 2 Department of Radiology, Hillel Yaffe Medical Center, Technion Faculty of Medicine, Hadera 38100, Israel; 3 Department of Internal Medicine, Hillel Yaffe Medical Center, Technion Faculty of Medicine, Hadera 38100, Israel; saif@hymc.health.gov.il; 4 Research Institute—Leumit Health Services, Tel-Aviv 6274411, Israel; aisrael@leumit.co.il; 5 Department of Epidemiology and Preventive Medicine, School of Public Health, Faculty of Medical & Health Sciences, Tel-Aviv University, Tel-Aviv 6274411, Israel

**Keywords:** chronic hepatitis B, metabolic dysfunction-associated steatotic liver disease, obesity, diabetes, dyslipidemia, HBeAg seroconversion, viral profile

## Abstract

**Background:** The concurrent presence of chronic hepatitis B virus (CHB) infection and metabolic dysfunction-associated steatotic liver disease (MASLD) presents a unique clinical scenario with implications that are not yet fully understood. This study aims to characterize the distinct clinical and virological features of CHB in the context of MASLD and evaluate its impact on disease progression and outcomes. **Methods:** Utilizing a comprehensive health maintenance organization database, this study included 1186 patients with CHB from 2000–2020. Patients were categorized into two groups: CHB-MASLD (*n* = 188) and CHB alone (*n* = 998). CHB diagnosis was confirmed by serological markers, while MASLD was diagnosed based on imaging and cardiometabolic risk factors. Comparative analysis and multiple regression models were applied to assess variables related to viral parameters and clinical outcomes. **Results:** The CHB-MASLD group was older (mean age of 45.2 vs. 39.1, *p* < 0.001) with higher rates of obesity (46.8% vs. 23.8%, *p* < 0.001), diabetes (36.2% vs. 17.3%, *p* < 0.001), and dyslipidemia. Distinct viral profiles included higher HBeAg negativity (96.2%), a higher rate of HBeAg-negative infection (70.4% vs. 63.8%; *p* < 0.001), and increased HBeAg seroconversion under treatment. Cirrhosis was more prevalent in the CHB-MASLD group (9.6% vs. 4.4%, *p* = 0.007), while HCC rates were comparable. Multivariate analysis identified age, male gender, chronic active hepatitis, and diabetes as predictors of cirrhosis. **Conclusions:** CHB-MASLD patients were distinguished by a higher prevalence of metabolic features, along with a distinct viral profile marked by increased chronic HBeAg infection, higher rates of HBeAg seroconversion, and a potential association with worse disease outcomes.

## 1. Introduction

Chronic hepatitis B virus (CHB) infection remains a considerable global health challenge, affecting more than 250 million individuals worldwide [1,2]. Simultaneously, non-alcoholic fatty liver disease (NAFLD), now recognized as metabolic dysfunction-associated steatotic liver disease (MASLD), has emerged as a leading metabolic disorder, establishing itself as the most prevalent chronic liver ailment, particularly in Western populations [3].

MASLD, characterized by a spectrum of liver disease stages ranging from simple steatosis to metabolic dysfunction-associated steatohepatitis (MASH), progressive fibrosis, and hepatocellular carcinoma (HCC), places a substantial burden on healthcare systems [4,5]. Concurrently, CHB infection assumes a pivotal role in the development of liver pathologies such as cirrhosis and hepatocellular carcinoma, contributing to increased liver-related mortality and morbidity [6].

The co-occurrence of CHB infection and MASLD is an emerging and complex phenomenon increasingly being encountered in daily practice [7]. This co-occurrence has raised important questions regarding the interactions between these two conditions, and has attracted significant research and clinical interest due to the potential for amplified liver injury progression and severe complications associated with both diseases.

While the exact mechanistic underpinnings linking CHB infection and the potential causative relationship between MASLD and CHB infection remain elusive, multiple hypotheses have been proposed [8,9,10]. Moreover, investigating the interplay between CHB and MASLD is an ongoing pursuit. Some findings have suggested that hepatic steatosis might have a favorable effect on CHB progression, possibly by accelerating hepatitis B surface antigen (HBsAg) serum clearance [11]. In addition, metabolic components and immune alterations related to MASLD progression have been suggested to directly inhibit HBV replication or indirectly induce anti-viral immune responses [12]. Conversely, MASLD may exacerbate liver damage in patients with chronic HBV infection, inducing oxidative stress, inflammation, and fibrosis [13]. Therefore, the coexistence of CHB infection and MASLD has been linked to an increased risk of advanced liver disease and HCC. However, several studies have reported inconclusive and conflicting results [14,15].

A comprehensive understanding of the intricate interplay between these two formidable liver diseases is paramount, as it forms the basis for designing therapeutic interventions that hold the potential to significantlyimprove clinical outcomes.

This study aims to investigate the clinical, viral, and biochemical characteristics of patients with concurrent CHB and MASLD, compared to CHB alone, and assess the impact of MASLD on liver disease progression, viral replication, and treatment outcomes.

## 2. Methods

### 2.1. Study Population

This study utilized the Leumit Health Service (LHS) database, a comprehensive repository of health-related records covering a vast cohort of 690,000 individuals from diverse ethnic backgrounds and geographical regions in Israel. The dataset encompasses records from 2000 to 2020, serving as the foundation for our research.

### 2.2. Patient Selection

Our study primarily focused on patients diagnosed with CHB to explore the complex interplay between MASLD and CHB. Eligible subjects were identified by including those with a confirmed CHB diagnosis indicated by appropriate ICD-10 codes and supported by serological markers indicative of chronic HBV infection. CHB was defined as the persistent presence of HBsAg or any evidence of replicative activity lasting for more than six months. Patients with HBsAg seroconversion within a six-month window, incomplete datasets, and those who had not undergone liver-focused imaging studies were excluded.

The diagnosis of MASLD relied on searching relevant ICD-10 codes for NAFLD (as no codes for MASLD are yet available), but was confirmed by the observation of hepatic steatosis in medical imaging, coupled with at least one cardiometabolic risk factor such as being overweight (BMI > 25 kg/m^2^), diagnosis of type 2 diabetes mellitus (T2DM), or treatment for T2DM, dyslipidemia (HDL < 40 mg/dL, triglycerides > 150 mg/dL, or use of lipid-lowering treatment), or hypertension (blood pressure > 130/85 or use of antihypertensive medications). Patients who had excessive alcohol consumption (defined as > 3 drinks per day) were excluded. Patients meeting these stringent criteria were categorized as the CHB-MASLD group, while those without evidence of MASLD in their medical imaging findings comprised the CHB-alone group.

### 2.3. Data Extraction

Relevant data were extracted from the LHS database, encompassing demographic details (age, sex, ethnicity, country of birth), body mass index (BMI) at the time of CHB diagnosis, clinical background, alcohol consumption habits, smoking status, and treatment history. Additionally, we documented the presence of relevant chronic liver diseases such as alcoholic liver disease, based on medical records, and chronic hepatitis C and hepatitis D co-infections based on serological results. Laboratory results, including hemoglobin, platelets, AST, ALT, bilirubin, INR, creatinine, ferritin, LDL, and triglycerides, were collected. Moreover, the CHB phase for each patient was documented and determined based on the available records of HBeAg status, ALT, HBV DNA values, and elastography or pathology results. Chronic active hepatitis was defined as chronic HBeAg positive or negative hepatitis with HBV DNA > 2000 IU/mL, ALT above the upper limit of normal (ULN), and/or at least moderate liver necroinflammation or fibrosis, as per the EASL guidelines [16].

### 2.4. Comparative Analysis

We compared various variables related to the CHB courses and outcomes between the CHB-MASLD and CHB-alone groups. Our analysis encompassed clinical and laboratory variables, particularly those related to the course of CHB and viral parameters.

We also investigated clinical outcomes, focusing on the development of advanced fibrosis, cirrhosis, and HCC during the follow-up period. Cirrhosis was identified using relevant ICD codes and confirmed through additional diagnostic methods, including liver biopsy, transient elastography or, in the absence of these tools, a combination of clinical, laboratory, and imaging data that provided a clear-cut picture of cirrhosis. HCC was identified based on ICD codes and confirmed via imaging modalities such as multiphase contrast-enhanced CT or MRI, in accordance with established diagnostic criteria. In cases where imaging alone was inconclusive, biopsy confirmation was utilized. The rate of advanced fibrosis was compared in both groups by calculating FIB-4 scores, based on the last available laboratory values, as close as possible to the end of the follow-up period. FIB-4 values > 2.65 were considered indicative of advanced liver fibrosis.

Finally, we conducted multivariate analyses to identify predictors influencing these critical outcomes, aiming to determine whether the presence of MASLD had any association with adverse clinical outcomes.

### 2.5. Ethical Approval

The study adhered to the principles outlined in the Helsinki Declaration and Rules of Good Clinical Practice. Approval for the study (26-19-ASF) was obtained from the Leumit Health Services (LHS) institutional review board. The LHS ethics committee waived the requirement for consent for data collection, analysis, and publication in this retrospective, non-interventional study, as all data were de-identified to safeguard patient privacy.

### 2.6. Statistical Analysis

Continuous variables were presented as the mean ± standard deviation, while categorical variables were expressed as percentages. Group differences were assessed using the t-test for continuous parameters and Fisher’s exact test for categorical variables.

For the multivariate analysis, a multiple multivariate logistic regression model adjusted for age, sex, ethnicity, and MASLD was employed to identify independent predictors of HBeAg seroconversion under treatment. For cirrhosis predictors, variables with significance at *p* < 0.05 or a notable trend at *p* < 0.1 were included in the multiple multivariate logistic regression model. SPSS version 25 facilitated these analyses, with the statistical significance set at *p* < 0.05.

## 3. Results

**Figure 1:**
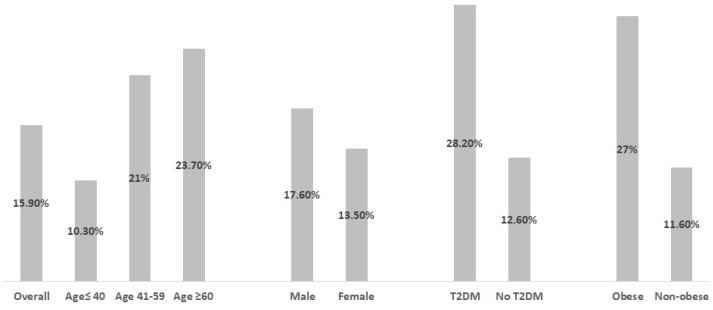
Prevalence of metabolic-associated steatotic liver disease (MASLD) in subpopulations of chronic hepatitis B (CHB) patients. The bar graph displays the prevalence of MASLD in various CHB subgroups based on age, sex, diabetes status, and obesity. Prevalence is represented as n (%), with a total cohort size of 1186 CHB patients. Subgroups include age ≤ 40 (n = 610), age 41–59 (n = 486), age ≥ 60 (n = 93); male (n = 697), female (n = 489); type 2 diabetes (T2DM) (n = 241), no T2DM (n = 945); obese (n = 326), non-Obese (n = 860). Abbreviations: MASLD, metabolic-associated steatotic liver disease; CHB, chronic hepatitis B; T2DM, type 2 diabetes mellitus.

The study cohort comprised almost 690,000 individuals. Among these, 1226 were diagnosed with CHB based on documented diagnoses and serologic evidence. Of these, 1186 CHB patients met the eligibility requirements and were divided into two groups: “CHB-MASLD” (*n* = 188) and the “isolated CHB” (*n* = 998) control group. The overall follow-up period was comparable for both groups, lasting 157.2 ± 58.5 months for CHB-MASLD patients and 148.4 ± 61.5 months for controls. Among the overall cohort of CHB patients, the diagnostic prevalence of MASLD displayed an ascending trajectory with increasing age, delineated as 10.3% for individuals below 40 years, 21.3% for those aged between 41 and 60, and 23.7% for those surpassing 60 years of age. Notably, the incidence demonstrated a discernible gender predilection, with a significantly higher occurrence among males at 17.4%, as opposed to 13.7% in females (*p* < 0.01). Additionally, the diagnostic rates were markedly elevated among individuals classified as obese (27% vs. 11.6%, *p* < 0.01) and those diagnosed with type 2 diabetes mellitus (T2DM) (28.2% vs. 12.6%, *p* < 0.001) (Figure 1). 

In the CHB-MASLD group, the mean age at diagnosis was significantly higher compared to the HBV non-MASLD group (45.2 vs. 39.1 years, *p* < 0.001).A predominance of male sex and Jewish ethnicity was observed in both groups, particularly in CHB-MASLD patients. The mean BMI index (30.6 ± 5.6 vs. 27.04 ± 5.04, *p* < 0.001), as well as the rate of obesity (BMI > 30; 46.8% vs. 23.8%, *p* < 0.001), were significantly higher in the CHB-MASLD group (Table 1).

The rate of HCV and HDV co-infections did not differ significantly between both groups and the rate of modest (<2 drinks/day) alcohol consumption was comparable (5.8% vs. 3.4%, *p* = 0.08). The CHB-MASLDgroup had a significantly lower percentage of current or past smokers (27.2% vs. 33.4%, *p* = 0.01). The prevalence of diabetes was significantly higher in the CHB-MASLD group compared to the controls (36.2% vs. 17.3%, *p* < 0.001).

The laboratory results (Table 2) highlighted notable variations in liver enzymes, iron metabolism, and lipid profiles. In the CHB-MASLD group, there were significantly higher levels of AST (median 35 vs. 31, *p* = 0.001), ALT (median 31 vs. 26, *p* < 0.001), ferritin (median 142.0 vs. 77.2, *p* < 0.001), and triglycerides (mean 147.7 vs. 115.6, *p* < 0.001).

Moreover, in the CHB-MASLD group, CHB phases and viral parameter distributions showed several significant differences compared to the isolated CHB patients (Table 3). Notably, the CHB-MASLD group had a significantly higher proportion of HBeAg-negative infection (70.4% vs. 63.8%, *p* < 0.001) and a lower prevalence of chronic active hepatitis (either HBeAg positive or negative) (29.5% vs. 35.5%, *p* = 0.002). Moreover, the CHB-MASLD patients had higher rates of overall HBeAg negativity. The on-treatment HBeAg seroconversion in these patients showed a trend toward significance, with 71.4% (5 out of 7) in the MASLD-CHB group compared to 44.9% (40 out of 89) in the CHB non-MASLD group (*p* = 0.04). In the multivariate analysis including age, sex, ethnicity, and MASLD, only MASLD (OR 1.21, 95 CI 1.12–1.34; *p* = 0.034) was a predictor of HBeAg seroconversion (Table 4).

In the context of CHB treatment, the analysis revealed no significant differences in treatment rates between the two groups. Specifically, 31.9% of patients with CHB-MASLD were having treatment compared to 32.4% of the control group (*p* = 0.73). Furthermore, there were no notable differences in the distribution of treatment regimens between the two groups. The types of treatment administered and their respective proportions are detailed in Table 3. 

At the conclusion of the follow-up, advanced fibrosis, indicated by a FIB-4 score > v2.65, was significantly more prevalent in the CHB-MASLD group (13.8%) compared to the isolated CHB group (6.2%, *p* < 0.001). Similarly, the incidence of cirrhosis was notably higher in the CHB-MASLD cohort (9.6%) than in the HBV non-MASLD group (4.4%, *p* = 0.007). However, the diagnosis rate of hepatocellular carcinoma (HCC) did not differ significantly between the two groups (0.5% vs. 0.9%, *p* = 1.00). 

In the multivariate analysis (Table 5), age at diagnosis maintained its statistical significance, revealing a substantial association with cirrhosis (*p* < 0.01, odds ratio [OR] 1.1, 95% confidence interval [CI]: 1.02–1.07). Similarly, the male gender, albeit with a slightly adjusted odds ratio, retained its significance in the multivariate model (*p* < 0.01, OR 1.2, 95% CI: 1.11–1.43). HCV co-infection and HDV AB positivity continued to exhibit significant associations with cirrhosis, with the latter demonstrating a particularly notable association (*p* < 0.01, OR 5.2, 95% CI: 1.32–19.15).Furthermore, chronic active hepatitis upheld a robust and independent association with cirrhosis, emphasizing its enduring significance in the multifaceted landscape of hepatic complications (*p* < 0.01, OR 9.1, 95% CI: 4.91–16.54).

Notably, T2DM sustained a considerable association with cirrhosis (*p* < 0.01, OR 1.8, 95% CI: 1.13–3.22), while MASLD and BMI did not manifest significant associations with cirrhosis in the multivariate analysis. 

## 4. Discussion

Investigations into the relationship between CHB and MASLD are ongoing, with an estimated prevalence of MASLD ranging from 15–30% among CHB patients [17,18]. In our cohort, concomitant CHB-MASLD was found in 15.4% of patients, with a higher MASLD diagnosis rate among older, male, obese, and diabetic CHB individuals, consistent with previous findings [19,20,21]. While our study was not designed to evaluate the prevalence of MASLD among CHB patients, the diagnosis rate appears significantly lower than in published data on MASLD prevalence in the general population [22,23]. Further evaluation is warranted, as accumulating evidence suggests that HBsAgseropositivity is associated with a lower risk of developing NAFLD, indicating a potential effect of HBV infection on NAFLD pathogenesis [24].

The current study revealed notable disparities in the CHB course within the CHB-MASLD cohort. The CHB-MASLD group exhibited a higher rate of HbeAg negativity (96.2%) compared to the isolated CHB group (89.7%). Additionally, the CHB-MASLD group showed a lower prevalence of chronic active hepatitis and a higher occurrence of HbeAg-negative infection, indicating a propensity towards an inactive carrier state. This altered viral profile in the context of metabolic comorbidity prompts deeper investigations into the mechanisms influencing HBV replication and the host immune response. In an HBV-immunocompetent mouse model scrutinizing the dynamics between HBV infection and hepatic steatosis, Hue et al. reported a noteworthy association where hepatic steatosis correlated with a substantial reduction in serum levels of HbeAg, hepatic hepatitis B core antigen (HbcAg), HbsAg, and HBV DNA. These outcomes suggest a potential affirmative influence of hepatic steatosis on the progression of CHB, possibly linked to the inhibition of HBV replication and proliferation [25]. However, further in-depth in vitro and in vivo investigations are essential to elucidate whether hepatic steatosis creates a local microenvironment that is suboptimal for HBV replication, or if the reduction in HBV replication is attributed to the systemic effects accompanying MASLD.

Of particular interest is the observation of a higher rate of HbeAg seroconversion under treatment in the CHB-MAFLD group, hinting at a nuanced interplay between metabolic factors and antiviral therapy. The impact of hepatic steatosis or MASLD on CHB treatment efficacy remains a subject of scholarly contention. While hepatic steatosis was found to be a baseline predictor of HbsAg clearance [26], several studies have suggested that hepatic steatosis is an independent factor for a poor response to direct-acting antiviral therapy, associated with lower rates of HBV-DNA suppression and a lower incidence of HbeAg loss [27,28]. Thus, the potential influence of metabolic alterations on treatment outcomes underscores the complexity of managing CHB-MASLD comorbidity, emphasizing the need for tailored therapeutic and follow-up approaches.

Despite the observed association between CHB coexisting with MASLD and the development of cirrhosis and advanced fibrosis, multivariate analysis identified age, male sex, chronic active hepatitis at presentation, and T2DM as independent predictors, yet failed to establish MASLD as a predictive factor for these advanced liver pathologies. This inconclusive determination can be attributed to the limited number of events and outcomes in the cohort, as well as the relatively young age of the study participants and the absence of detailed histological data. Consequently, definitive conclusions regarding the specific impact of MASLD on the clinical course and outcomes of CHB remain elusive. Furthermore, the lack of comprehensive data on the treatment status of MASLD patients, coupled with the well-established knowledge that proper treatment of CHB significantly reduces the risk of cirrhosis and HCC, further complicates the establishment of MASLD as an independent predictor for these outcomes. Several studies have also shown no correlation between steatosis and fibrosis [29,30] in CHB patients. Chang et al. followed treatment-naïve CHB patients with normal ALT and HBV DNA < 2000 IU/mL for up to 3 years and demonstrated no correlation between hepatic steatosis and fibrosis progression [31]. Thus, whether metabolic factors combined with virologic factors play a greater role in the progression of liver fibrosis, and for a more nuanced understanding of the histopathological spectrum and its implications on the progression and outcomes of CHB-MASLD in coexistence, long-term, large-sample studies incorporating liver biopsies or advanced non-invasive assessment tools for liver fibrosis are warranted in thefuture.

Clinicians should be aware of the distinct clinical and metabolic features of patients with concurrent CHB and MASLD, as these have significant implications for patient care. Despite higher rates of HbeAg-positive chronic infection and HbeAg seroconversion, the metabolic profiles of these patients, characterized by obesity, diabetes, and dyslipidemia, may predispose them to worse outcomes. These findings emphasize the need for an integrated approach addressing both viral and metabolic factors. Clinicians should prioritize lifestyle interventions, weight management, glycemic control, and lipid-lowering therapies to mitigate the overall burden of liver disease. Further research is required to determine whether stricter or more frequent monitoring, such as with FibroScan or elastography, or earlier implementation of HCC surveillance, is warranted.

It is noteworthy that despite efforts to ensure the study’s validity by exclusively considering new cases during the study period and maintaining comparable follow-up times between the CHB and CHB-MAFLD groups, certain limitations and challenges may have influenced the results. The inability to conduct a time-dependent analysis is one such limitation.While the study took measures to address confounding variables, the complex and dynamic nature of liver diseases could introduce variations over time that were not accounted for in the analysis. Additionally, as this is a retrospective study based on data from a database, certain important data, which could elucidate the impact of MASLD on chronic HBV infections, were not available, potentially limiting the ability to fully assess this relationship. Furthermore, it is crucial to acknowledge that the study’s sample size and events may have posed as constraints on drawing definitive conclusions, particularly regarding the development of HCC. Moreover, the lack of liver biopsies underscores the challenge of precisely characterizing the spectrum of liver pathology within CHB-MASLD coexistence.

In conclusion, this study offers a comprehensive characterization of patients with concurrent CHB and MASLD. Our findings emphasize the distinct clinical and viral profiles of CHB-MASLD patients, particularly the heightened prevalence of metabolic risk factors such as obesity, diabetes, and dyslipidemia. This group also exhibits a unique viral profile, with notably increased rates of hBeAg-negative infection and higher hBeAg seroconversionundertreatment. Furthermore, the co-occurrence of MASLD appears to correlate with a potentially elevated risk of cirrhosis, underscoring the exacerbating effect of metabolic dysfunction on liver disease progression in this population. These results suggest that MASLD may act as a critical modifier in the clinical trajectory of CHB, which calls for tailored management approaches. Future research should focus on therapeutic strategies that concurrently target both viral and metabolic factors to optimize clinical outcomes in this increasingly prevalent dual-disease context.

## Figures and Tables

**Table 1:** Baseline characteristics of the CHB-MASLD and isolated CHB groups.

Variable (N/%)	CHB-MASLD (N = 188)	Isolated CHB (N = 998)	*p* Value
Age at diagnosis	45.2 ± 12.5; [17–75]	39.1 ± 14.2; [17–89]	<0.01
≤40	63 (33.5%)	547 (54.8%)	<0.01
41–59	103 (54.8%)	380 (38.1%)	
60≤	22 (11.7%)	71 (7.1%)	
Sex (male)	121 (64.4%)	576 (57.7%)	0.09
Ethnicity (Jewish)	131 (69.7%)	634 (63.5%)	0.02
Country of birth (Israel) %	174 (92.6%)	961 (96.3%)	0.03
Follow up duration (months)	157.2 ± 58.5	148.4 ± 61.5	0.07
BMI > 30	88 (46.8%)	238 (23.8%)	<0.01
BMI (mean)	30.6 ± 5.6	27.04 ± 5.04	<0.01
HCV co-infection	7 (3.7%)	57 (5.7%)	0.38
HDV (Ab positive)	5 (3.1%)	50 (6.1%)	0.14
Modest alcohol consumption	11 (5.8%)	34 (3.4%)	0.08
Current or past smoker	49 (27.2%)	305 (33.4%)	0.01
Type 2 diabetes (%)	68 (36.2%)	173 (17.3%)	<0.01
Hypertension (%)	35 (18.6%)	177 (17.7%)	0.15
Dyslipidemia (%)	60 (31.9%)	201 (20.1%)	<0.01

Abbreviations: BMI, body mass index; HCV, hepatitis C virus; HDV, hepatitis D virus; MASLD, metabolic-associated steatotic liver disease; CHB, chronic hepatitis B.

**Table 2:** Comparison of major laboratory results between the CHB-MASLD and isolated CHB groups.

Variable	CHB-MASLD (N = 188)	Isolated CHB (N = 998)	*p* Value
Hemoglobin (g/dL)	14.1 ± 1.58	13.93 ± 1.72	0.13
Platelets (×10^3^/μL)	209.7 ± 56.4	212.8 ± 59.4	0.52
Aspartate aminotransferase (U/L)	35 [19.3–89.0]	31 [19–92]	<0.01
Alanine aminotransferase (U/L)	31 [22–97]	26 [18–99]	<0.01
Ferritin (ng/mL)	[181.5–50.1] 142.0	[130.4–36.4] 77.2	<0.01
Bilirubin (g/dL)	0.56 [0.44–0.78]	0.59 [0.41–0.81]	0.97
Creatinine (mg/dL)	0.88 ± 0.62	0.81 ± 0.41	0.07
Triglycerides (mg/dL)	147.7 ± 84.8	115.6 ± 71.5	<0.01
Low-density lipoprotein(mg/dL)	111.9 ± 32.07	110.1 ± 34.4	0.48
International normalized ratio (INR)	1.01 ± 0.14	1.03 ± 0.34	0.36

Abbreviations: MASLD, metabolic-associatedsteatotic liver disease; CHB, chronic hepatitis B.

**Table 3:** Viral parameters, chronic hepatitis B infection course, and patients’ outcomes in both groups.

Variable n(%)	CHB-MASLD (N = 188)	Isolated CHB (N = 998)	*p* Value
CHB Phase			
Full data on CHB phase	156 (82.3%)	886 (88.8%)	N/A
HBeAg+ infection HBeAg+ hepatitis HBeAg– infection HBeAg– hepatitis	0 (0.0%) 7 (3.8%) 131 (70.4%) 48 (25.8%)	6 (0.7%) 83 (9.4%) 565 (63.8%) 232 (26.2%)	0.121 0.002 <0.001 0.181
Active hepatitis (HBeAg −/+)	55 (29.5%)	315 (35.5%)	0.002
HBeAg seroconversion	5(71.4%)	40 (44.9%)	0.041
CHBtreatment (%)	60 (31.9%)	324 (32.5%)	0.932
Treatment type			
Treatment Entecavir Tenfovir Lamivudin Cedofovir	60 (31.9%) 23 (12.2%) 21 (11.2%) 15 (8.0%) 1 (0.5%)	324 (32.4%) 119 (11.9%) 91 (9.1%) 109 (10.9%) 5 (0.5%)	0.73
Patients’ Outcome			
FIB-4 Index > 2.65	26 (13.8%)	62 (6.2%)	<0.001
Cirrhosis	18 (9.6%)	44 (4.4%)	0.007
Hepatocellular carcinoma	1 (0.5%)	9 (0.9%)	1.00

Abbreviations: MASLD, metabolic-associated steatotic liver disease; CHB, chronic hepatitis B.

**Table 4:** Predictors of under-treatment HBeAg seroconversion, a multivariate analysis.

Variables	*p*-Value	Odds ratio	95% C.I.	
			Upper	Lower
Age (years)	0.178	1.431	0.978	2.118
Sex (male)	0.961	1.044	0.128	3.634
Ethnicity (Jewish)	0.727	0.927	0.567	1.425
Metabolic-associated steatotic liver disease	0.034	1.21	1.121	1.344

**Table 5:** Predictors for liver cirrhosis in chronic HBV patients, amultivariate analysis.

	Univariate Analysis				Multivariate	
Study Group	Without Cirrhosis	Cirrhosis	*p*-Value	OR (95%CI)	*p*-Value	OR (95%CI)
Age at diagnosis	39.7 ± 14	48.03 ± 13.5	<0.01	1.15 (1.1–1.3)	<0.01	1.1 (1.02–1.07)
Gender (male)	648 (58%)	49 (79%)	<0.01	1.3 (1.1–1.4)	<0.01	1.2 (1.11–1.43)
Body mass index	27.1 ± 4.4	27.6 ± 5.3	0.12			
Smoking	337 (32.6%)	17 (28.8%)	0.82			
HCV Co-infection	45 (4.5%)	13 (21.0%)	<0.01			
HDV AB positive	47 (4.9%)	10 (18.9%)	<0.01	5.1 (2.6–10)	<0.01	5.2 (1.32–19.15)
MASLD	171 (15%)	18 (24.3%)	0.031	1.8 (1.1–3.2)	0.43	1.3 (0.71–2.33)
T2DM	218 (19.4%)	23 (37.1%)	<0.01	2.8 (1.7–4.6)	<0.01	1.8 (1.13–3.22)
Chronic active hepatitis	446 (43.7%)	52 (98%)	<0.01	4.55 (2.4–8.2)	<0.01	9.1 (4.91–16.54)

Abbreviations: HCV, hepatitis C virus; HDV, hepatitis D virus; MASLD, metabolic-associated steatotic liver disease; T2DM, type 2 diabetes mellitus.

## Data Availability

The data that support the findings of this study are available on request from the corresponding author. The data are not publicly available due to restrictions from LHS as it contains information that could compromise the privacy of research participants.
